# A miniaturized MEMS accelerometer with anti-spring mechanism for enhancing sensitivity

**DOI:** 10.1038/s41378-024-00826-x

**Published:** 2025-03-05

**Authors:** Ruihong Xiong, Xuankai Xu, Yushuai Liu, Shihao Du, Lihui Jin, Fang Chen, Tao Wu

**Affiliations:** 1 School of Information Science and Technology, ShanghaiTech University, 201210 Shanghai, China; 2 State Key Lab of Transducer Technology, Shanghai Institute of Microsystem and Information Technology, Chinese Academy of Sciences, 200050 Shanghai, China; 3 University of Chinese Academy of Sciences, 100049 Beijing, China; 4 Shanghai Engineering Research Center of Energy Efficient and Custom AI IC, 201210 Shanghai, China

**Keywords:** Sensors, Electrical and electronic engineering

## Abstract

Anti-spring mechanisms are widely used for improving the noise performance of MEMS accelerometers due to their stiffness softening effect. However, the existing mechanisms typically require large bias force and displacement for achieving stiffness softening, leading to large device dimensions. Here, we propose a novel anti-spring mechanism composed of two pre-shaped curved beams connected in a parallel configuration, which can achieve stiffness softening without requiring large bias force and displacement. The stiffness softening effect of the mechanism is verified through theoretical modeling and finite element method (FEM) simulation. After that, the mechanism is implemented in a 4.2 mm × 4.9 mm MEMS capacitive accelerometer prototype. The experimental results reveal that the sensitivity of the accelerometer increases by 10.4% compared to the initial sensitivity; at the same time, the noise floor and bias instability decrease by 10.5% and 4.2%. The sensitivity, nonlinearity, bias instability, and noise floor after biasing are 51.1 mV/g, 0.99%, 0.24 mg, and 21.3 ${\rm{\mu }}{\rm{g}}/\sqrt{{\rm{Hz}}}$, respectively. Thus, the proposed mechanism can enhance the performance of the accelerometer. This work provides an innovative approach for improving the performance of MEMS accelerometers while enabling miniaturization.

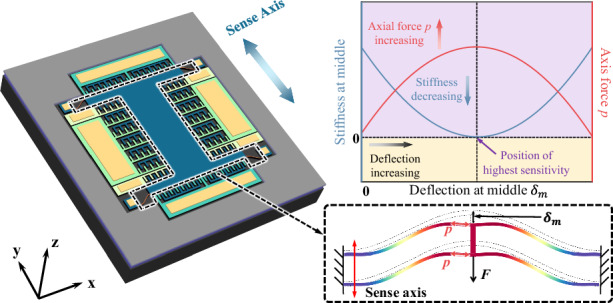

## Introduction

High-resolution microelectromechanical systems (MEMS) accelerometers are essential in a variety of applications, including gravity measurement^1^, inertial navigation^2^, oil detection^3^, earthquake monitoring^4^, and structure monitoring^5^. The resolution of MEMS accelerometers is limited by the noise floor, which is determined by calculating the total noise equivalent acceleration^6^. Consequently, improving the noise floor significantly is essential for achieving high-resolution measurements. Several approaches have been explored for MEMS accelerometers to improve the noise floor, which can be broadly categorized into two main strategies: reducing input-referred noise and enhancing sensitivity. Methods for reducing input-referred noise involve integrating a large inertial proof mass^7–11^, reducing damping coefficient^12–14^, and utilizing low noise interface circuit^15–17^. Additionally, the sensitivity can be enhanced by developing novel mode-localization sensing mechanisms^18–21^, optical sensing schemes^22,23^, and reducing stiffness constant^24–27^.

Recently, a novel method based on the anti-spring effect has been applied to enhance the sensitivity of MEMS accelerometers for achieving a low noise floor, which leads to several designs in quasi-zero stiffness anti-spring mechanisms. An anti-spring mechanism exhibits a unique mechanical behavior where its stiffness decreases with increasing displacement^6^. This is achieved by introducing an axial compression force. As the displacement occurs, this compression force generates a counteracting force that cancels part of the restoring force introduced by the spring, thus effectively reducing the total stiffness. The existing anti-spring mechanisms typically require compression or pre-loading forces for introducing a compression force, such as those provided by an active actuation system^28^, or earth gravity^29^. Geometric anti-spring (GAS) usually consists of two sets of arched flexures that meet at a constrained central point and become increasingly compliant as displacement grows^30^. Boom et al.^31^ were the first to integrate GAS into a MEMS accelerometer as an anti-spring mechanism by designing an on-chip mechanical preloading system. This system longitudinally compresses the GAS to induce axis force and reduce the lateral stiffness, and its complexity and size lead to a bulky structure of the accelerometer.

Middlemiss et al.^32^ developed a MEMS accelerometer based on an anti-spring mechanism composed of a GAS and an arched cantilever, achieving a noise floor of 40 ${\rm{ng}}/\sqrt{{\rm{Hz}}}$. This is the first MEMS accelerometer that can measure earth tides. Based on this work, the same research group^33^ later developed a field-portable MEMS accelerometer with a noise floor of 8 ${\rm{ng}}/\sqrt{{\rm{Hz}}}$ using the previously reported anti-spring mechanism^32^. EI Mansouri et al.^34^ developed a MEMS accelerometer based on an anti-spring mechanism composed of two sets of GAS, with a noise floor of 17 ${\rm{ng}}/\sqrt{{\rm{Hz}}}$. In addition to the anti-spring mechanisms achieved through GAS, Tu et al.^29,35^ developed a MEMS accelerometer based on an anti-spring mechanism composed of a curved bistable beam and two folded beams, achieving a noise floor of 8 ${\rm{ng}}/\sqrt{{\rm{Hz}}}$. This design utilizes the negative stiffness of the bistable beam to counteract the positive stiffness of the folded beams, effectively reducing the overall stiffness to near zero. All these anti-spring mechanisms were pre-loaded using the gravity of the proof mass within the accelerometers. However, these anti-spring mechanisms^29,32–35^ require a large bias force, necessitating a large proof mass structure for pre-loading. This requirement leads to large dimensions for the accelerometer device (the order of 10^−2^ m), which can result in difficulty, high cost, and low yield in fabrication.

In this paper, a novel anti-spring mechanism that can achieve stiffness softening without requiring large bias force and displacement is proposed, modeled, and analyzed. The proposed anti-spring mechanism is composed of two clamped-clamped pre-shaped curved beams, each with the same dimensions, connected in a parallel configuration to a rigid shuttle at their middle. The stiffness softening effect of the proposed anti-spring mechanism is verified through both a theoretical model and FEM simulation. The comparison shows excellent agreement between the theoretical results and the FEM results. The most significant advancement of this novel anti-spring mechanism is its reduced requirements for large bias force and displacement; the required bias force and displacement of the proposed anti-spring mechanism are more than an order of magnitude lower than the existing designs composed of three or four-arched beams connected in parallel^32–34^. This reduction eliminates the requirement of a large proof mass, which effectively reduces the device size.

Furthermore, we developed a miniaturized MEMS capacitive accelerometer prototype that integrates this novel anti-spring mechanism to demonstrate its practical application. The electrostatic force produced by the capacitive comb fingers is used as the bias force. The experiments demonstrate efficient performance enhancement of the accelerometer prototype after electrostatically biasing. Compared to the accelerometers reported in refs. ^32–34^, the accelerometer with the proposed anti-spring mechanism is significantly more compact, with the die size reduced by at least an order of magnitude. This miniaturization is a crucial advancement, allowing for broader application flexibility and ease of integration into various systems. Additionally, the installation requirements are substantially reduced as the design eliminates the need for gravitational biasing, which typically requires precise orientation. This work introduces an innovative method that enhances the performance of MEMS accelerometers while also enabling miniaturization.

In the “Results” section, the modeling and geometry optimization of the anti-spring mechanism are theoretically investigated. Additionally, the implementation of this mechanism in a miniaturized accelerometer is introduced. In the “Discussion” section, the required improvements for MEMS accelerometers to achieve a lower stiffness and reduced noise floor, further enhancing the overall performance, are introduced. In the “Methods” section, the mathematical derivation, the detailed fabrication processes, and the experimental setup are introduced.

## Results

### Modeling and design of the anti-spring mechanism

Bistable mechanisms, composed of elastic beams or membranes, are widely employed across various MEMS applications, including microrobotics^36^, switch^37^, resonators^38^, sensors^39,40^, and actuator^41^, owing to their simplicity, low actuation energy, large stroke with small restoring forces, and snap-through behavior. Among these, beam-type bistable mechanisms are particularly prevalent in MEMS as compliant mechanisms, favored for their straightforward structure and negative stiffness properties. The beam-type bistable mechanism composed of two buckled-like curved beams connected at their middle has significant force-deflection nolinearity^42,43^. During deflection, the axis force occurs due to axial compression, reaching a maximum at the position where the beam is compressed to its minimum length. If the max axis force exceeds a critical load, the beam exhibits bistable behavior.

**Fig. 1:**
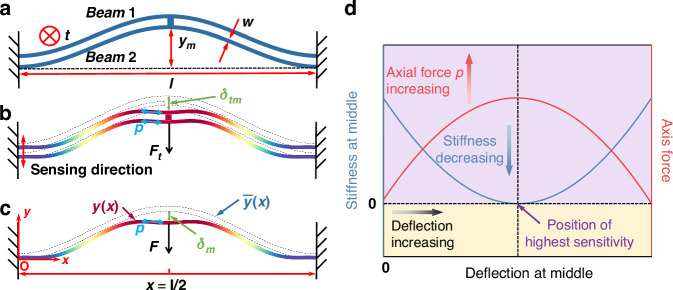
The proposed anti-spring mechanism. **a** Schematic of the proposed anti-spring mechanism with dimension definitions. **b** Schematic of the proposed anti-spring mechanism after deflection. **c** Schematic of a clamped-clamped pre-shaped curved beam in the initial position and after deflection under a lateral force *F* applied to its middle. **d** Typical axis force and stiffness curves for the proposed anti-spring mechanism. High sensitivity can be achieved by biasing the curved beams at the critical state of buckling using electrostatic force

The proposed anti-spring mechanism consists of two clamped–clamped pre-shaped curved beams, which have the same dimensions and are connected in parallel to a rigid shuttle at their middle, its initial shape is designed as the first buckling mode shape, and each of the curved beams in the anti-spring mechanism has a uniform shape, which includes depth $t$, width $w$, span $l,$ and initial middle height ${y}_{{\rm {m}}}$, as shown in Fig. 1a. The dimension of the curved beam satisfies the small deformation hypothesis ($w\ll l,{y}_{{\rm {m}}}\ll l$). When the two curved beams deflect under a lateral force *F*_*t*_ applied to their middle, they are compressed, giving rise to an axial compression force *p* within each beam, as shown in Fig. 1b. As the middle deflection of the two curved beams ${\delta }_{{{\rm {tm}}}}$ increases, the beams become more compressed, leading to an increase in the axis force, canceling part of the restoring force introduced by the curved beam in the sensing direction, consequently reducing the effective stiffness, as shown in Fig. 1d. Our work is inspired by the bistable mechanism reported in reference^42,43^. On this basis, we optimized the geometry parameters of the buckled-like curved beam to ensure that the maximum axial force generated during deflection remains at the critical load, thereby achieving extremely low stiffness at the critical state of buckling. The stiffness of the anti-spring mechanism in the sensing direction can be reduced by biasing the beam at a specific position using electrostatic force, as shown in Fig. 1d, thereby enhancing the accelerometer sensitivity.

**Fig. 2:**
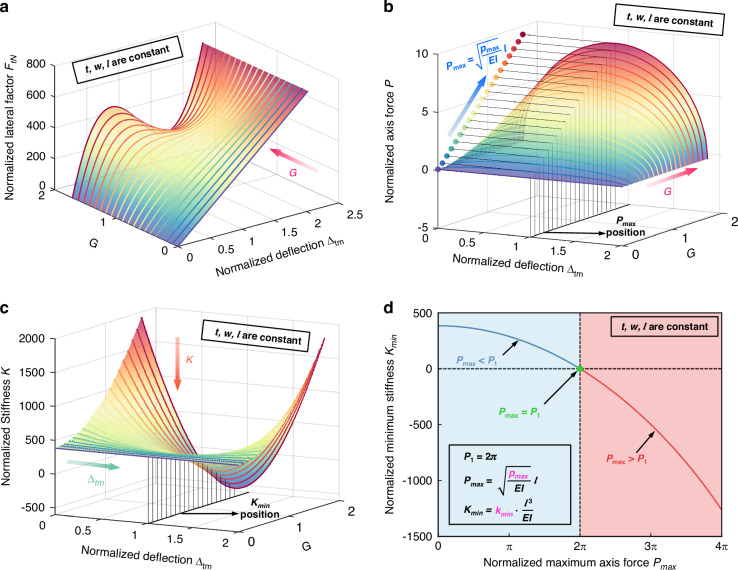
Characteristic curves from theoretical modeling. **a** Normalized force–deflection curve for the anti-spring mechanism. **b** Normalized axis force–deflection curves for the anti-spring mechanism. **c** Normalized stiffness–deflection curves for the anti-spring mechanism. The curves are shown considering the anti-spring mechanisms with different *G* while keeping *t*, *w*, and *l* constant. **d** Normalized minimum stiffness–maximum axis force curves for the anti-spring mechanism

Since the two clamped–clamped pre-shaped curved beams are identical in dimensions and connected in parallel to a rigid shuttle at their middle, the mechanical behavior of the anti-spring mechanism can be established by modeling one of the curved beams. Considering a single curved beam deflected under a lateral force $F$ applied to its middle, and its middle deflection is ${\delta }_{{\rm {m}}}$, as shown in Fig. 2c. A cartesian coordinate system is used, and the origin is located at the fixed end on the left side of the single curved beam. The as-fabricated shape and deformed shape of the curved beam after deflection are described by $\bar{y}\left(x\right)$ and $y\left(x\right)$, respectively. The as-fabricated shape is

1 $$\bar{y}\left(x\right)=\frac{{y}_{{\rm {m}}}}{2}\left(1-\cos 2\pi \frac{x}{l}\right)$$

The height-to-width ratio $G$ is critical to the mechanical behavior of the curved beam^42^, and it is defined as

2 $$G=\frac{{y}_{{\rm {m}}}}{w}$$

The mechanical behavior of the single curved beam is governed by the following system of equations:

3 $$F=\frac{{\left(\frac{p{l}^{2}}{{EI}}\right)}^{\frac{3}{2}}}{\frac{{l}^{3}}{{EI}}\left(\sqrt{\frac{p{l}^{2}}{16{EI}}}-\tan \left(\sqrt{\frac{p{l}^{2}}{16{EI}}}\right)\right)}\left(\frac{{y}_{{\rm {m}}}p}{\left(p-{p}_{1}\right)}-{\delta }_{{\rm {m}}}\right)$$

4 $$\frac{3l}{16{y}_{{\rm {m}}}^{2}{p}^{2}}\left(1-\frac{\tan \left(\sqrt{\frac{p{l}^{2}}{16{EI}}}\right)}{\sqrt{\frac{p{l}^{2}}{16{EI}}}}+\frac{{\tan }^{2}\left(\sqrt{\frac{p{l}^{2}}{16{EI}}}\right)}{3}\right){F}^{2}-\frac{{p}_{1}}{{{y}_{{\rm {m}}}\left(p-{p}_{1}\right)}^{2}}F+\frac{l\left(2p{p}_{1}^{2}-{p}^{2}{p}_{1}\right)}{16{EI}{\left(p-{p}_{1}\right)}^{2}}+\frac{pl}{12{G}^{2}{EI}}=0$$

where *E* is Young’s modulus, $I$ is the moment of inertia of the single curved beam, and ${p}_{1}$ is the 1st critical buckling load, which is calculated as ${p}_{1}=\frac{4{\pi }^{2}{EI}}{{l}^{2}}$ according to Eq. (21) in the “Methods” section. And the mathematical derivation of Eqs. (3) and (4) are presented in the “Methods” section.

Since the two curved beams are connected in parallel to a rigid shuttle at their middle, the middle defection of the anti-spring mechanism ${\delta }_{{{\rm {tm}}}}$ is the same as the deflection of the single curved beam ${\delta }_{{\rm {m}}}$, and the lateral force applied to the middle of single curved beam *F* is half of the lateral force applied to the middle of the anti-spring mechanism *F*_t_, which are expressed as follows:

5 $${\delta }_{{{\rm {tm}}}}={\delta }_{{\rm {m}}};{F}_{{\rm {t}}}=2F$$

By substituting Eq. (5) into Eqs. (3), and (4), the system of equations governing the mechanical behavior of the anti-spring mechanism can be derived as follows:

6 $${F}_{{\rm {t}}}=2\frac{{\left(\frac{p{l}^{2}}{{EI}}\right)}^{\frac{3}{2}}}{\frac{{l}^{3}}{{EI}}\left(\sqrt{\frac{p{l}^{2}}{16{EI}}}-\tan \left(\sqrt{\frac{p{l}^{2}}{16{EI}}}\right)\right)}\left(\frac{{y}_{{\rm {m}}}p}{\left(p-{p}_{1}\right)}-{\delta }_{{{\rm {tm}}}}\right)$$

7 $$\frac{3l}{64{y}_{{{m}}}^{2}{p}^{2}}\left(1-\frac{\tan \left(\sqrt{\frac{p{l}^{2}}{16{EI}}}\right)}{\sqrt{\frac{p{l}^{2}}{16{EI}}}}+\frac{{\tan }^{2}\left(\sqrt{\frac{p{l}^{2}}{16{EI}}}\right)}{3}\right){F}_{t}^{2}-\frac{{p}_{1}}{{2{y}_{{\rm {m}}}\left(p-{p}_{1}\right)}^{2}}{F}_{t}+\frac{l\left(2p{p}_{1}^{2}-{p}^{2}{p}_{1}\right)}{16{EI}{\left(p-{p}_{1}\right)}^{2}}+\frac{pl}{12{G}^{2}{EI}}=0$$

Figure 2 shows the theoretical characteristic curves for the anti-spring mechanism where depth $t$, width $w$, and span $l$ remain constant, while the height-to-width ratio *G* varies from 0 to 1.8. The curves include the normalized lateral force–deflection (*F*_tN_−Δ_tm_), axial force–deflection (*P*–Δ_tm_), and stiffness–deflection (*K*−Δ_tm_) curves, respectively. The variables $P$, ${\Delta }_{{{\rm {tm}}}}$, ${F}_{{{\rm {tN}}}}$, and *K* are the normalized axial force, the normalized deflection, the normalized lateral force, and the normalized stiffness, respectively, and are defined as follows:

8 $$P=\sqrt{\frac{p}{{EI}}}l;{\Delta }_{{{tm}}}=\frac{{\delta }_{{{tm}}}}{{y}_{{m}}};{F}_{{{tN}}}=\frac{{F}_{{t}}{l}^{3}}{{EI}{y}_{{m}}};K=\frac{k{l}^{3}}{{EI}}$$

As shown in Fig. 2a, with an increase in the height-to-width ratio *G*, the linear force–deflection relationship of the anti-spring mechanism gradually transitions to a nonlinear force–deflection relationship during deflection. This implies that the lateral stiffness of the anti-spring mechanism $k$ varies during deflection, which is defined as $\frac{\partial {F}_{{\rm {t}}}}{\partial {\delta }_{{{tm}}}}$. During deflection, the axial force $p$ increases from zero, while the stiffness $k$ decreases from an initial value determined by the dimensions. Both the axial force and stiffness reach their respective maximum (denoted by ${p}_{max }$) and minimum (denoted by ${k}_{min }$) near a position where the ${\delta }_{{{tm}}}$ equals ${y}_{m}$, as shown in Fig. 2b, c. Therefore, to reduce the lateral stiffness of the anti-spring mechanism and enhance the sensitivity of the accelerometer, the operating point is selected at the position of the minimum stiffness (the position of maximum axial force). We can calculate the stiffness of the anti-spring mechanism at the operating point as follows:

9 $${k}_{min }={\left(\frac{\partial {F}_{{t}}}{\partial {\delta }_{{{tm}}}}\right)}_{p={p}_{max }}=-2\frac{{\left(\frac{{p}_{max }{l}^{2}}{{EI}}\right)}^{\frac{3}{2}}}{\frac{{l}^{3}}{{EI}}\left(\sqrt{\frac{{p}_{max }{l}^{2}}{16{EI}}}-\tan \left(\sqrt{\frac{{p}_{max }{l}^{2}}{16{EI}}}\right)\right)}$$

We can find that ${k}_{min }$ is zero for ${P}_{max }={P}_{1}$ (${p}_{max }={p}_{1}$), positive for ${P}_{max } < {P}_{1}$ (${p}_{max } < {p}_{1}$), and negative for ${P}_{max } > {P}_{1}$ (${p}_{max } > {p}_{1}$), as shown in Fig. 2d.

The height-to-width ratio *G* determines the value of ${p}_{max }$, which subsequently dictates the lateral stiffness of the anti-spring mechanism at the operating point, as shown in Fig. 2b, d. According to the above analysis, we can find a height-to-width ratio ${G}_{z}$ that satisfies the condition where ${p}_{\max }$ equals ${p}_{1}$, which can theoretically maximize the stiffness softening of the anti-spring mechanism at the operating point. As can be seen from Eq. (28) in the “Methods” section, ${p}_{\max }$ cannot equal ${p}_{1}$ unless the following equation holds, as follows:

10 $$-\frac{2{F}_{z}}{l}+\frac{{y}_{{\rm {m}}}{p}_{1}^{2}}{2{EI}}=0$$

where ${F}_{z}$ is the lateral force for the anti-spring mechanism at the operating point when ${p}_{\max }={p}_{1}$. ${G}_{z}$ can be obtained by substituting ${F}_{{t}}={F}_{z}=\frac{{y}_{{m}}l{p}_{1}^{2}}{4{EI}}$, $F=\frac{{F}_{t}}{2}$, and $p={p}_{\max }={p}_{1}$ into Eq. (33) in the “Methods” section, as follows:

11 $${G}_{z}=\sqrt{\frac{{p}_{1}}{12\left(\frac{{p}_{1}}{16}-\sum _{j=5,9\ldots }\frac{{p}_{1}^{4}}{{16p}_{j}{\left({p}_{1}-{p}_{j}\right)}^{2}}\right)}}\approx \sqrt{\frac{{p}_{1}}{12\left(\frac{{p}_{1}}{16}-\frac{{p}_{1}^{4}}{{16p}_{5}{\left({p}_{1}-{p}_{5}\right)}^{2}}\right)}}=1.155$$

where ${p}_{j}$ is the *j*th critical buckling load, determined by Eq. (21) in the “Methods” section.

For the anti-spring mechanism with a height-to-width ratio ${G}_{z}$, the bias displacement ${\delta }_{{\rm {b}}}$ and bias force ${F}_{{{\rm {bs}}}}$ are calculated as follows:

12 $${\delta }_{{b}}\approx {y}_{{m}}={G}_{z}w;{F}_{{{bs}}}={F}_{z}={\pi }^{4}E{G}_{z}\frac{{w}^{4}t}{3{l}^{3}}$$

To reduce the bias force and displacement of the anti-spring mechanism, we should reduce the width and depth of the curved beam, and increase the span of the curved beam as much as possible. The width $w$, depth $t,$ and span $l$ of the curved beam are designed as 2 μm, 25 μm, and 500 μm, respectively. The final design parameters of the anti-spring mechanism are listed in Table 1.

**Table 1:** The dimensions of the anti-spring mechanism

Symbol	Meaning	Value
*G*	Height-to-width ratio	1.155
*w*	Width of the curved beam	2 μm
*t*	Depth of the curved beam	25 μm
*l*	Span of the curved beam	500 μm

**Fig. 3:**
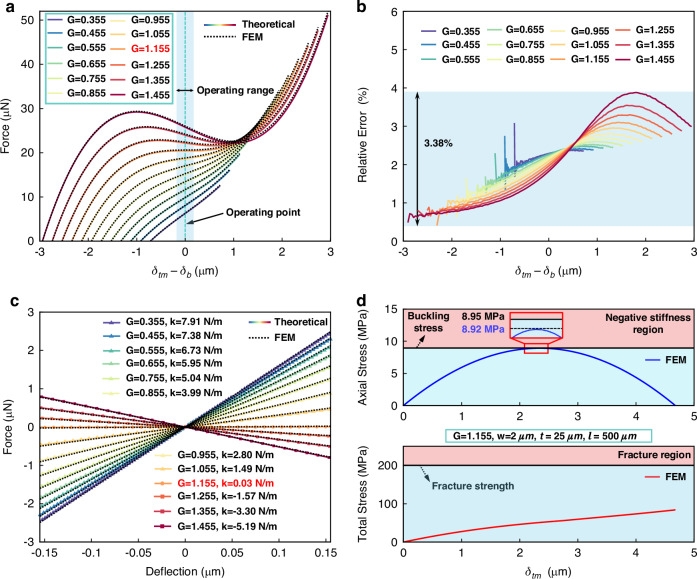
Comparison between characteristic curves obtained from theoretical modeling and FEM simulation. **a** Force–deflection curves for the anti-spring mechanisms, obtained from theoretical modeling and FEM simulation. **b** Relative error curves between force–deflection curves obtained from theoretical modeling and FEM simulation. **c** Stiffness variation of the anti-spring mechanisms at the operating point, obtained from theoretical modeling and FEM simulation. The curves consider the anti-spring mechanisms with a width of 2 μm, a depth of 25 μm, a span of 500 μm, and different *G* varying from 0.355 to 1.455. **d** Axial stress–deflection curve and total stress-deflection curve for the anti-spring mechanism with a width of 2 μm, a depth of 25 μm, a span of 500 μm, and a *G* of 1.155, obtained from FEM simulation

The theoretical model of the anti-spring mechanism presented above is verified through FEM using COMSOL. For the case that the anti-spring mechanism with a width of 2 μm, a depth of 25 μm, a span of 500 μm and different *G* varying from 0.355 to 1.455, the force-deflection curves for the anti-spring mechanism obtained theoretically and with FEM simulation are shown in Fig. 3a. For better comparison, the force-deflection curves are shifted with ${\delta }_{{\rm {tm}}} -{\delta }_{{\rm {b}}}$ to maintain the operating point to zero. The stiffness of the proposed anti-spring mechanism near the operating point decreases as *G* increases, exhibiting a significant transition from positive to negative stiffness, as shown in Fig. 3a. The maximum relative error between theoretical curves and FEM curves is 3.8%, as shown in Fig. 3b, indicating excellent agreement between the theoretical model and the FEM model of the anti-spring mechanism. Figure 3c shows the stiffness variations (operating range is 0.2 μm) of the anti-spring mechanism with different height-to-width ratios at the operating point, obtained from theoretical modeling and FEM simulation, respectively. The stiffness of the anti-spring mechanism will soften from 8.01 to 0.13 N/m when $G$ increases from 0.355 to 1.155, according to FEM, which can significantly enhance the sensitivity of the accelerometer.

During deflection, the stress inside each curved beam of the anti-spring mechanism includes axial stress due to axial compression and bending stress due to bending. According to the above analysis, the critical buckling stress can be calculated as follows:

13 $${\sigma }_{p1}=\frac{{p}_{1}}{{wt}}$$

**Fig. 4:**
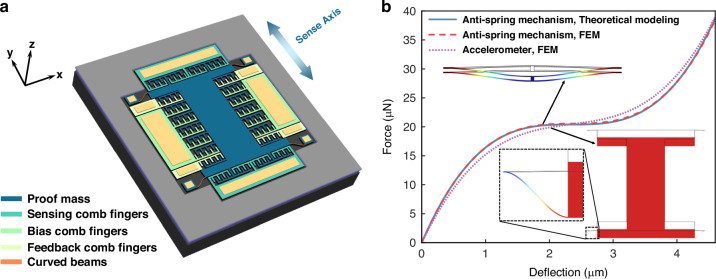
Schematic of a miniaturized capacitive accelerometer with the proposed anti-spring mechanism and its characteristic curves. **a** A miniaturized accelerometer using the proposed anti-spring mechanism as a suspension structure. **b** Comparison between force–deflection curves for the accelerometer and the proposed anti-spring mechanism. The anti-spring mechanism curves are obtained from theoretical modeling and FEM simulation, while the accelerometer curves are obtained from FEM simulation

According to Eq. (13), the buckling stress of the anti-spring mechanism with design parameters listed in Table 1, is 8.95 MPa. The axial stress-deflection curve and total stress-deflection curve for this anti-spring mechanism, obtained from FEM simulation, are shown in Fig. 4d. During deflection, the maximum axial stress is 8.92 MPa, lower than the critical buckling stress. Additionally, the total stress increases monotonically with deflection, reaching its maximum of 84.1 MPa at the end of deflection, which is significantly lower than the fracture strength of single crystal silicon^44^ (200 MPa). Thus, this anti-spring mechanism can operate safely without the risk of buckling or fracture failure during deflection.

### Implementation of the anti-spring mechanism in a miniaturized MEMS accelerometer

The basic principle of an accelerometer is that it measures acceleration by detecting the displacement of a proof mass or the stress on suspension beams caused by inertial forces. According to the displacement or stress transduction mechanisms, MEMS accelerometers can be classified into several types: capacitive^45,46^, optical^47^, resonant^48^, and piezoelectric^49^. capacitive accelerometers are widely used due to the advantages of high sensitivity, good temperature stability, low noise, and low power consumption.

The proposed anti-spring mechanism is integrated into a capacitive accelerometer to demonstrate its practical application. A miniaturized capacitive MEMS accelerometer with the anti-spring mechanism is shown in Fig. 4a. A proof mass is suspended by two curved beams anchored to a fixed frame, used for responding to inertial forces generated by acceleration, thus deforming the curved beams and moving the proof mass in the sensing direction. The resulting displacement of the proof mass is detected by the capacitance change of two sets of differential area-changing comb fingers, which have better linearity than gap-changing comb fingers. A set of bias capacitors composed of area-changing comb fingers are on both sides of the proof mass, generating electrostatic force for biasing the anti-spring mechanism. Two sets of feedback capacitors, also composed of area-changing comb fingers, are on both sides of the proof mass. Although not used in this work, these feedback comb fingers can be used for implementing closed-loop operations in future works. The minimum gap size of the comb fingers is 2 μm, which is designed for better sensing and actuation sensitivity. The geometry parameters of the accelerometer are listed in Table 2.

**Table 2:** The dimensions of the proposed accelerometer

Parameters	Value
Width of the proof mass	930 μm
Length of the proof mass	2450 μm
Capacitor gap	2 μm
Total mass	138 μg
Die area	4.2 mm × 4.9 mm

The force–deflection curves for the proposed anti-spring mechanism and the miniaturized MEMS accelerometer are shown in Fig. 4b. In theory, the connection of the curved beams will constrain the lateral deflection at the middle to a single linear degree of freedom (DOF) along the *y*-axis, which can fully constrain the even-order buckling modes in ${\delta }_{{{\rm {tm}}}}$. However, there are some differences between the FEM-simulated curves for the accelerometer and the theoretical curves for the anti-spring mechanism. This is because the slight rotation of the middle of the curved beam during the accelerometer operation causes the even-order buckling modes to not be fully constrained.

In this work, the electrostatic force generated by the area-changing comb fingers is used as the bias force. Due to the bias voltage, the total stiffness softening of the accelerometer is the result of the combined effects of mechanical spring softening and electrostatic spring softening. The stiffness of the accelerometer at the operating point must remain positive. In practical applications of the anti-spring mechanism, the position with a bias displacement of 1.6 μm is chosen as the operating point for the accelerometer to satisfy this requirement. At the operating point, the calculated stiffness softening of the accelerometer due to the electrostatic spring softening, based on the electrostatic force modeling in ref. ^50^, is −3.5 N/m. According to the FEM simulation results, the stiffness softening of the accelerometer due to the mechanical spring softening is −19 N/m (stiffness decreases from 22.7 to 3.7 N/m) with a bias force of 18.8 μN and a bias displacement of 1.6 μm. Therefore, the total stiffness softening of the accelerometer is −22.5 N/m. Based on the above analysis, the stiffness of the accelerometer softens from 22.7 to 0.2 N/m with an electrostatic bias force of 18.8 μN and a bias displacement of 1.6 μm, this indicates that this anti-spring mechanism has great potential to significantly enhance the sensitivity of the MEMS accelerometer.

### Experiments of the fabricated accelerometer

**Fig. 5:**
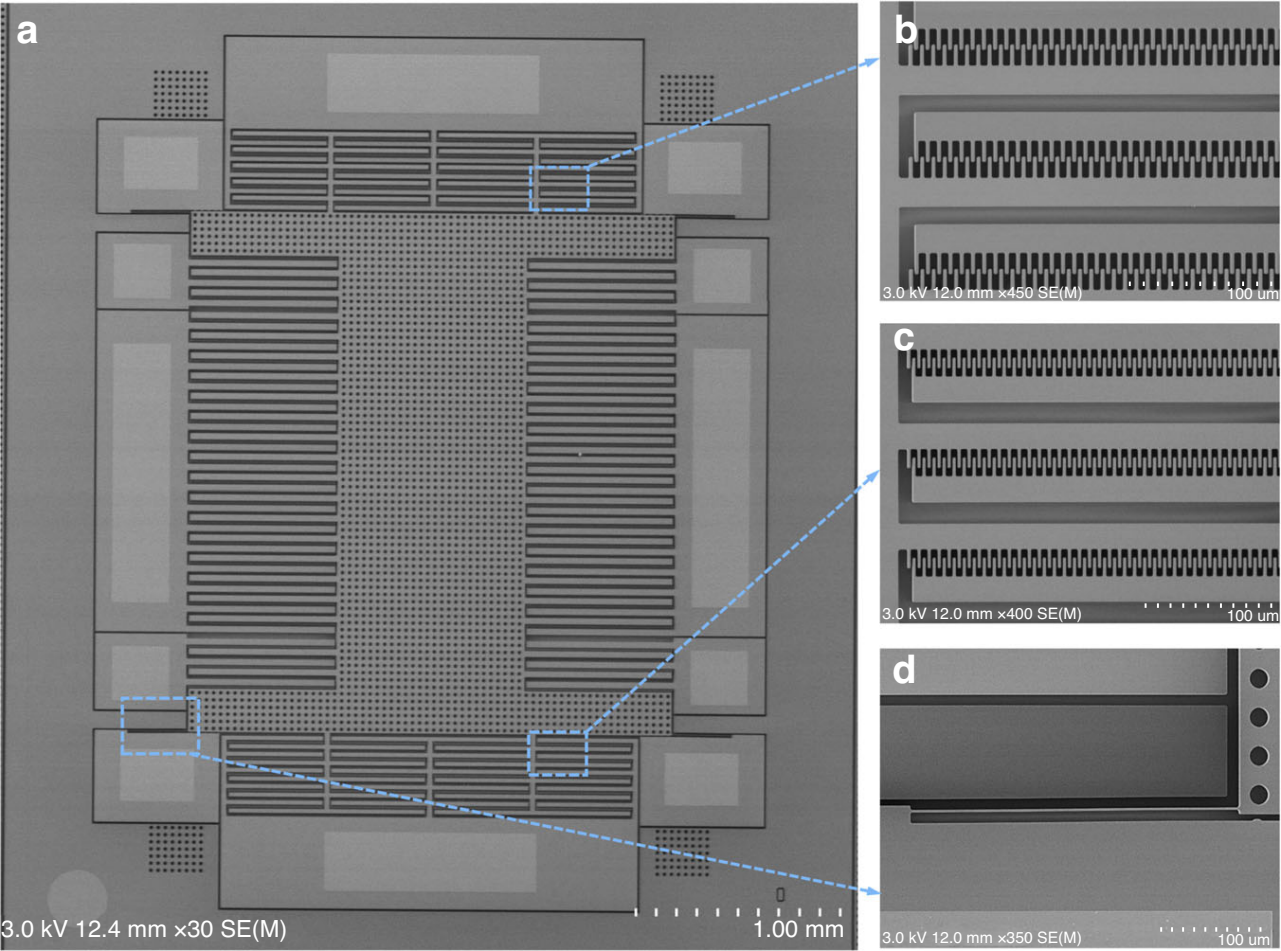
SEM picture of the accelerometer die. **a** The fabricated accelerometer die. **b** and **c** Zoomed-in view of the sensing comb drives. **d** Zoomed-in view of half of the single curved beam in the anti-spring mechanism

To demonstrate its practical application for an accelerometer, we integrate the proposed anti-spring mechanism into a capacitive accelerometer. Figure 5a shows the SEM picture of the fabricated accelerometer dies before dicing. The external dimensions of the die are only 4.2 mm × 4.9 mm. Figure 5b, c show the zoomed-in view of the sensing comb drives, and the fabricated overlapping length of the two sets of differential area-changing sensing comb fingers is different due to the necessity of a bias displacement before operating. The fabricated overlapping length of the bias comb fingers is the same as in Fig. 5b. The proof mass is suspended by two curved beams, and half of the single curved beam is shown in Fig. 5d. The mechanical stopper near the end of the curved beams will prevent structure failure due to overload.

**Fig. 6:**
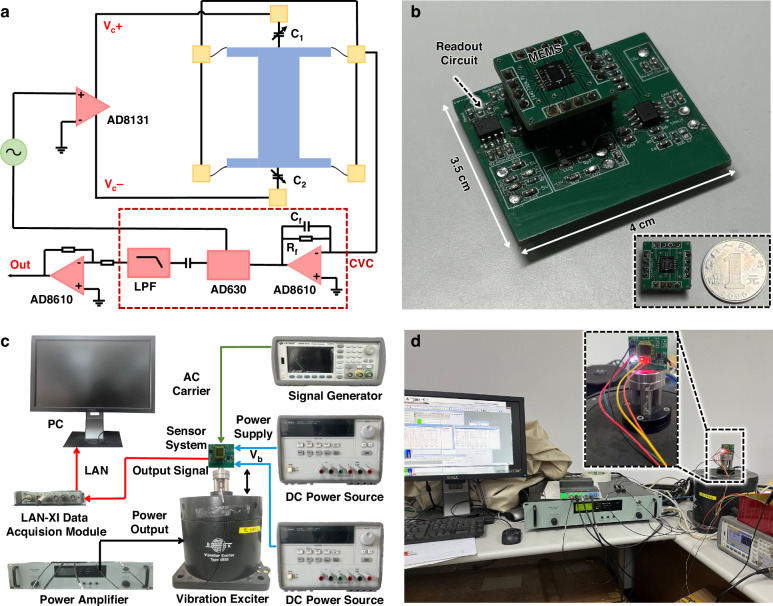
Experimental principle. **a** Schematic diagram of the readout circuit for the accelerometer. **b** Experimental setup of the readout circuit board with simply packaged MEMS die. **c** Schematic diagram of the experiment setup. **d** Experiment setup

These experiments were conducted to demonstrate the effectiveness of the proposed anti-spring mechanism to enhance the sensitivity of the miniaturized accelerometer prototype. Figure 6a shows the schematic diagram of the readout circuit for the accelerometer, which includes a capacitance-to-voltage converter (CVC) and a voltage amplifier. The readout circuit is implemented on a standard printed circuit board (PCB), the MEMS die after dicing is simply packaged on a PCB carrier and wire bonded with gold wires. The packaged accelerometer is assembled on the readout circuit PCB. The experimental setup of the readout circuit board with a simply packaged MEMS die is shown in Fig. 6b. The total size of the readout circuit board is 3.5 cm × 4 cm. The schematic diagram of the test setup to characterize the performance of the accelerometer is shown in Fig. 6c, and the readout circuit PCB with the simply packaged accelerometer is mounted to a position stage on a commercial shaker (B&K 3629), which can provide the acceleration with controllable amplitude and frequency. The experiment setup is shown in Fig. 6d.

**Fig. 7:**
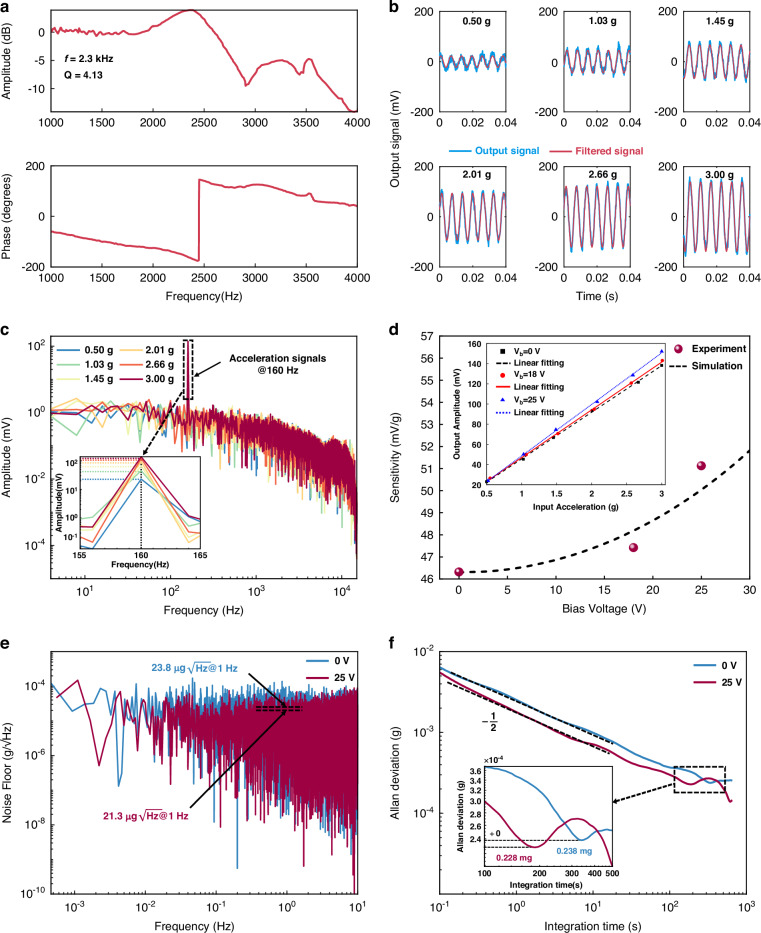
Performance of the MEMS accelerometer. **a** Measured frequency response of the accelerometer. **b** Measured time domain output signals and filtered signals. **c** FFT spectrums of the output signals of the accelerometer without bias voltage. **d** The measured sensitivities and simulated sensitivities of the accelerometer under different bias voltages. **e** Noise floor of the accelerometer with *V*_b_ = 0 V and *V*_b_ = 25 V, respectively. **f** Allan deviation of the accelerometer with *V*_b_ = 0 V and *V*_b_ = 25 V, respectively. All the measurements are conducted under the open-loop test condition

The characterizations of the accelerometer are conducted under atmosphere pressure (1 atm) at room temperature (25 °C). The frequency response of the accelerometer at the output is shown in Fig. 7a. The accelerometer is mounted to a position stage on the shaker and excited using a commercial shaker. As can be seen, the resonance frequency of the accelerometer is 2.3 kHz, and the corresponding *Q* (quality factor) is about 4.13. The sensitivity and nonlinearity of the accelerometer are assessed by subjecting it to sinusoidal accelerations ranging from 0.5 *g* to 3 *g* (at 160 Hz) using a shaker. The time-domain output and filtered signals of the accelerometer without bias voltage (*V*_b_) are shown in Fig. 7b, and the amplitude of the output signals is extracted from the corresponding FFT spectrum, as illustrated in Fig. 7c. The original sensitivity of the accelerometer (*V*_b_ = 0) is 46.3 mV/g with a nonlinearity of 1.02%.

To verify the feasibility of the proposed anti-spring mechanism in enhancing the sensitivity of the accelerometer, we vary *V*_b_ to apply different bias forces, setting different operating points for the accelerometer. The measured sensitivity variations under different bias voltages are determined by measuring the amplitude variations of the output signals with input accelerations ranging from 0.5 *g* to 3 *g*, as shown in Fig. 7d. The measured sensitivity of the accelerometer with different bias voltages aligns with the simulated sensitivity. When *V*_b_ is applied to the accelerometer, its sensitivity increases. For *V*_b_ = 25 V applied to the accelerometer, the sensitivity is 51.1 mV/g enhanced by 10.4% compared to the original sensitivity and the nonlinearity is 0.99%. This indicates that the anti-spring mechanism effectively enhances the sensitivity of the accelerometer.

Static outputs of the accelerometer, without any active temperature control, are sampled at 800 Hz for over 30 min to characterize the output stability and noise floor of the accelerometer. The noise floors of the accelerometer with *V*_b_ = 0 V and *V*_b_ = 25 V are compared, the noise floor of the accelerometer is down from 23.8 to 21.3 ${\rm{\mu }}{\rm{g}}/\sqrt{{\rm{Hz}}}$ at 10 Hz after biasing, as shown in Fig. 7e. The Allan deviations of the accelerometer with *V*_b_ =0 V and *V*_b_ = 25 V are compared, and the bias instability of the accelerometer is down from 0.238 to 0.228 mg after biasing, as shown in Fig. 7f. Since a low output noise voltage source is used to provide the bias voltage, its noise is negligible compared to other electronic noise, indicating that the output noise of the accelerometer is almost unaffected by the noise of the power source. The noise equivalent acceleration and bias instability of the accelerometer are improved due to the sensitivity enhancement.

## Discussion

The measurement results prove the feasibility of the proposed anti-spring mechanism to enhance the sensitivity of MEMS accelerometers, as well as, improve the noise floor and bias instability. Theoretically, by providing a stable and high-precision bias force to bias the accelerometer at its designed operating point, it is possible to achieve near-zero stiffness, thereby enabling unlimited sensitivity. However, the measured sensitivity enhancement is different from the designed sensitivity enhancement, which means there is a deviation between the actual operating point and the designed operating point. There are two potential reasons for this difference: the effect of fabrication tolerances (such as sidewall verticality of the curved beam) is not considered in the theoretical model; and the applied voltage is lower than required to achieve the designed bias force, which results in a difference between the actual electrostatic force and the designed bias force. More accurate modeling of the anti-spring mechanism, an increase in the applied voltage, and optimization of fabrication tolerances could further improve sensitivity.

Our proposed anti-spring mechanism enables implementation in a miniaturized MEMS accelerometer while enhancing sensitivity. Compared to MEMS accelerometers with other anti-spring mechanisms reported in refs. ^32–34^, our MEMS accelerometer achieves at least an order of magnitude reduction in die size, which can allow for broader application flexibility and ease of integration into various systems. However, our MEMS accelerometer has lower sensitivity and limited resolution due to the smaller proof mass weight and sensing capacitance compared to the existing accelerometers with other anti-spring mechanisms. The proof mass weight of our prototype is only 138 μg, whereas the proof mass weight in ref. ^34^ is 42.48 mg. To further enhance the performance of our accelerometer, several improvements can be implemented, external environmental conditions can impact the resolution of capacitive accelerometers, especially in high-precision measurement applications. The resolution of the accelerometer can be further improved by employing an industrial-grade ceramic package to effectively integrate and package the fabricated die. Furthermore, optimizing the interface circuits and experimental setup can lead to increased sensitivity and reduced noise floors. Specifically, using ASIC for signal extraction and processing is expected to result in higher resolution. Conducting measurements in environments far from noise sources would further enhance the resolution of the accelerometer.

## Conclusion

This paper presents a novel anti-spring mechanism to enhance the sensitivity of MEMS accelerometers while enabling miniaturization. The proposed mechanism, consisting of two clamped-clamped pre-shaped curved beams connected in a parallel configuration, can achieve stiffness softening with small bias force and displacement, as verified through theoretical modeling and FEM simulations. Implementing this mechanism in a capacitive MEMS accelerometer prototype demonstrates a 10.4% increase in sensitivity, a 10.5% decrease in noise floor, and a 4.2% reduction in bias instability. The compact size (4.2 mm × 4.9 mm) of the prototype highlights its potential for broader application flexibility and ease of integration. Future work will focus on optimizing fabrication tolerances, adopting a new packaging method, and improving the interface circuits to further enhance performance. This innovative approach offers a pathway to improving the performance of MEMS accelerometers while enabling miniaturization.

## Methods

### Mathematical derivation

This section presents the mathematical derivation for solving the static equilibrium equation of the single curved beam and the system of equations governing the mechanical behavior of the single curved beam, the important parameters for the mathematical derivation are calculated as follows:

14 $$\delta \left(x\right)=\bar{y}\left(x\right)-y\left(x\right)$$

15 $$\Delta {\rm{s}}\approx \frac{1}{2}\mathop{\int }\limits_{0}^{l}\left({\left(\frac{d\bar{y}}{{dx}}\right)}^{2}-{\left(\frac{dy}{{dx}}\right)}^{2}\right){dx}$$

16 $$p={Ebt}\left(\frac{\Delta {s}}{\bar{s}}\right)$$

where $\Delta {s}$ is the change in the total length of the curved beam after deflection, and $\bar{s}$ is the initial total length of the curved beam.

Based on the classical elasticity theory, the static equilibrium equation of the single curved beam subjected to a lateral force $F$ and an axial force $p$ can be derived as

17 $${EI}\frac{{{\rm {d}}}^{4}\delta }{{{{\rm {d}}x}}^{4}}+p\frac{{{\rm {d}}}^{2}\delta }{{\rm {d}}{x}^{2}}=-\frac{4F}{l}\sum _{j=1,5,9\ldots }\cos \left(\frac{{N}_{j}x}{l}\right)+p\frac{{{\rm {d}}}^{2}\bar{y}}{{\rm {d}}{x}^{2}}$$

where ${N}_{j}$ is a set of constants $\left({N}_{j}=\left(j+1\right)\pi ,j=\mathrm{1,5,9}\ldots \right)$. When a specific lateral force $F$ is applied to the middle of the curved beam, the resulting deflection $\delta$ and axial force *p* are determinate. As a result, the static equilibrium equation forms a fourth-order linear non-homogeneous differential equation with constant coefficients. The solution can be obtained by finding the general solution ${\delta }_{{g}}$ and a particular solution ${\delta }_{{p}}$. The corresponding homogeneous equation for Eq. (17) is

18 $${EI}\frac{{{\rm {d}}}^{4}{\delta }_{{g}}}{{{{\rm {d}}x}}^{4}}+p\frac{{{\rm {d}}}^{2}{\delta }_{g}}{{\rm {d}}{x}^{2}}=0$$

which is the same equation for determining the critical buckling loads of a straight beam with the same dimensions. With clamped-clamped conditions (${\delta }_{{{g}}}\left(0\right)={\delta }_{{{g}}}\left(l\right)=0,\frac{{\rm {d}}{\delta }_{{{g}}}}{{{\rm{d}}x}}\left(0\right)=\frac{{{\rm {d}}}{\delta }_{{{g}}}}{{{\rm {d}}x}}\left(l\right)=0$), $p$ must meet the equation as follows to have nontrivial solutions.

19 $$\sin \left(\frac{l}{2}\sqrt{\frac{p}{{EI}}}\right)\cdot \left(\tan \left(\frac{l}{2}\sqrt{\frac{p}{{EI}}}\right)-\frac{l}{2}\sqrt{\frac{p}{{EI}}}\right)=0$$

After solving Eq. (20), we can get different solutions that meet the boundary conditions, as follows

20 $$\left\{\begin{array}{l}{\delta }_{j}\left(x\right)=1-\left(\cos \sqrt{\frac{{p}_{j}}{{EI}}}\,x\right)\qquad\qquad\qquad\qquad j=1,3,5\ldots \\ {\delta }_{j}\left(x\right)=1-\cos \left(\sqrt{\frac{{p}_{j}}{{EI}}}\,x\right)-2\frac{x}{l}+\frac{2\sin \left(\sqrt{\frac{{p}_{j}}{{EI}}}\,x\right)}{\sqrt{\frac{{p}_{j}}{{EI}}}l}j=2,4,6\ldots \\ \,\end{array}\right.$$

and

21 $$\left\{\begin{array}{l}\sqrt{\frac{{p}_{j}}{{EI}}}l=\left(j+1\right)\pi\qquad\qquad\qquad\qquad j=1,3,5\ldots \\ \sqrt{\frac{{p}_{j}}{{EI}}}l=2.86\pi ,4.92\pi ,6.94\pi \ldots\qquad j=2,4,6\ldots \end{array}\right.$$

where ${p}_{j}$ is the *j*th critical buckling load, ${\delta }_{j}$ is the *j*th buckling mode.

The solution of Eq. (18) can be assumed as the superposition of the buckling modes as follows^42^

22 $${\delta }_{g}\left(x\right)=\sum _{j=1,2,3\ldots }{{m}_{j}\delta }_{j}\left(x\right)$$

where ${m}_{j}$ is the superposition coefficient of the *j*th buckling mode in the general solution ${\delta }_{g}$. To determine ${m}_{j}$, substituting Eq. (22) into Eq. (18) derives the following equation:

23 $$\mathop{\sum}\limits_{j=1,2,3\ldots }{p}_{j}\left(p-{p}_{j}\right){m}_{j}\cos \left(\sqrt{\frac{{p}_{j}}{{EI}}}\,x\right)=0$$

According to Eq. (23), ${m}_{j}$ equals zero unless $p$ is identical to ${p}_{j}$. For cases where $p$ equals ${p}_{j}$, ${m}_{j}$ can take any value. The connection of the curved beams constrains the non-symmetrical buckling modes in the general solution, implying that ${m}_{j}$ is zero for even values of $j$. In summary, the values of ${m}_{j}$ are determined as follows:

24 $$\left\{\begin{array}{l}{m}_{j}\in {\mathbb{R}}\qquad\qquad p={p}_{j},j=1,3,5,\ldots \\ {m}_{j}=0\qquad\qquad p\ne {p}_{j},j=1,3,5,\ldots \\ {m}_{j}=0\qquad\qquad\qquad\quad j=2,4,6,\ldots \end{array}\right.$$

Next, we need to find a particular solution ${\delta }_{p}$ for solving the static equilibrium equation. The particular solution ${\delta }_{{p}}$ satisfies the equation as follows:

25 $${EI}\frac{{{{d}}}^{4}{\delta }_{{{p}}}}{{{{{d}}x}}^{4}}+p\frac{{{{d}}}^{2}{\delta }_{{{p}}}}{{{d}}{x}^{2}}=-\frac{4F}{l}\sum _{j=1,5,9\ldots }\cos \left(\frac{{N}_{j}}{l}x\right)+p\frac{{{{d}}}^{2}\bar{y}}{{{d}}{x}^{2}}$$

According to Eqs. (20) and (21), ${N}_{j}$ satisfies that ${N}_{j}=\sqrt{\frac{{p}_{j}}{{EI}}}{l}$ in Eq. (25). Consequently, a particular solution has a form ${{n}_{j}\delta }_{j}\left(x\right)$ can satisfy the clamped–clamped condition for each term $\cos \left(\frac{{N}_{j}x}{l}\right)$ in Eq. (25). So, the particular solution ${\delta }_{{\rm {p}}}$ can also be assumed as the superposition of the buckling modes as follows:

26 $${\delta }_{{{p}}}\left(x\right)=\sum _{j=1,2,3\ldots }{{n}_{j}\delta }_{j}\left(x\right)$$

where ${n}_{j}$ is the superposition coefficient of the *j*th buckling mode in the particular solution ${\delta }_{{p}}$. The equation to determine ${n}_{j}$ is derived by substituting Eqs. (1), (20), and (26) into Eq. (25) as follows:

27 $$\begin{array}{l}\mathop{\sum}\limits_{j=1,2,3\ldots }{n}_{j}\left(\frac{{pp}_{j}}{{EI}}-\frac{{p}_{j}^{2}}{{EI}}\right)\cos \left(\sqrt{\frac{{p}_{j}}{{EI}}}\,x\right)\\\qquad=\,-\frac{4F}{l}\mathop{\sum}\limits_{j=1,5,9\ldots }\left(\cos \left(\sqrt{\frac{{p}_{j}}{{EI}}}x\right)\right)+\frac{{y}_{{{m}}}}{2}\frac{p{p}_{1}}{{EI}}\cos \left(\sqrt{\frac{{p}_{1}}{{EI}}}x\right)\end{array}$$

The coefficient of each term $\cos \scriptstyle{\left(\sqrt{\frac{{p}_{j}}{{EI}}}x\right)}$ on both sides of Eq. (27) needs to be the same. Therefore, ${n}_{j}$ are determined as follows:

28 $$\left\{\begin{array}{l}{n}_{j}=\frac{\left(-\frac{4F}{l}+\frac{{y}_{{{m}}}p{p}_{j}}{2{EI}}\right){EI}}{{p}_{j}\left(p-{p}_{j}\right)}\qquad\qquad j=1\\ {n}_{j}=-\frac{\frac{4F}{l}{EI}}{{p}_{j}\left(p-{p}_{j}\right)}\qquad\qquad\qquad j=5,9\ldots \\ {n}_{j}=0\qquad\qquad\qquad\qquad j=3,7,11\ldots \\ {n}_{j}=0\qquad\qquad\qquad\qquad j=2,4,6\ldots \end{array}\right.$$

After determining ${m}_{j}$ and ${n}_{j}$, the deflection $\delta$ at various positions along the beam $x$ can be expressed as follows:

29 $$\delta \left(x\right)=\sum _{j=1,2,3\ldots }{{(m}_{j}+{n}_{j})\delta }_{j}\left(x\right)$$

where ${m}_{j}$ and ${n}_{j}$ are determined by Eqs. (24) and (28), respectively. We focus on the deflection of the beam in the middle ${\delta }_{{\rm {m}}}$, which represents the displacement of the proof mass under acceleration inputs and is calculated as follows:

30 $${\delta }_{{{m}}}=\delta \left(\frac{l}{2}\right)=\mathop{\sum}\limits _{j=1,2,3\ldots }{({m}_{j}+{n}_{j})\delta }_{j}\left(\frac{l}{2}\right)$$

In this paper, we focus exclusively on the case where the curved beam is in the compressible phase, where the axial force $p$ is always below the 3rd critical buckling load ${p}_{3}$^43^. According to Eqs. (28) and (30), ${\delta }_{{\rm {m}}}$ is related to $F$ and $p.$ The equations describing the relationship between $F$, $p$ and ${\delta }_{{\rm {m}}}$ can be derived by the follows derivation.

To calculate $p$, we need to calculate, the initial total length of the beam $\bar{s}$ and the change in the total length of the curved beam after deflection $\Delta {s}.\,\bar{s}$ is calculated as follows:

31 $$\bar{s}=l+\frac{{y}_{{{m}}}^{2}l{p}_{1}}{16{EI}}$$

where $\Delta {s}$ is calculated by substituting Eqs. (1), (14), (24), (28), and (29) into Eq. (15), as follows:

32 $$\Delta s=\sum _{j=1,5,9\ldots }\frac{4{EI}}{{{lp}}_{j}{\left(p-{p}_{j}\right)}^{2}}{F}^{2}-\frac{{{y}_{{{m}}}p}_{1}}{{\left(p-{p}_{1}\right)}^{2}}F+\frac{{y}_{{{m}}}^{2}l\left(2p{p}_{1}^{2}-{p}^{2}{p}_{1}\right)}{16{EI}{\left(p-{p}_{1}\right)}^{2}}$$

$p$ is calculated by substituting Eqs. (31), and (32), into Eq. (16), and after simplification, the equation describing $F$–$p$ relationship of the single curved beam can be obtained as follows:

33 $$\sum _{j=1,5,9\ldots }\frac{4{EI}}{{{y}_{{{m}}}^{2}{lp}}_{j}{\left(p-{p}_{j}\right)}^{2}}{F}^{2}-\frac{{p}_{1}}{{{y}_{{{m}}}\left(p-{p}_{1}\right)}^{2}}F+\frac{l\left(2p{p}_{1}^{2}-{p}^{2}{p}_{1}\right)}{16{EI}{\left(p-{p}_{1}\right)}^{2}}+\frac{pl}{12{G}^{2}{EI}}=0$$

The equation describing the $F$–${\delta }_{{\rm {m}}}$ relationship of the single curved beam is calculated by substituting Eqs. (24) and (28) into Eq. (30), as follows:

34 $$F=\frac{1}{\sum _{j=1,5,9\ldots }\frac{8{EI}}{l{p}_{j}\left(p-{p}_{j}\right)}}\left(\frac{{y}_{{{m}}}p}{\left(p-{p}_{1}\right)}-{\delta }_{{{m}}}\right)$$

Infinite series in Eqs. (33) and (34) complicate the numerical solution of these equations. Following the simplification of the infinite series in Eqs. (33) and (34) as detailed in ref. ^43^, the system of equations governing the mechanical behavior of the single curved beam (the relationship between $F$, $p$ and ${\delta }_{{\rm {m}}}$) is expressed as follows:

35 $$F=\frac{{\left(\frac{p{l}^{2}}{{EI}}\right)}^{\frac{3}{2}}}{\frac{{l}^{3}}{{EI}}\left(\sqrt{\frac{p{l}^{2}}{16{EI}}}-\tan \left(\sqrt{\frac{p{l}^{2}}{16{EI}}}\right)\right)}\left(\frac{{y}_{{m}}p}{\left(p-{p}_{1}\right)}-{\delta }_{{{m}}}\right)$$

36 $$\frac{3l}{16{y}_{{{m}}}^{2}{p}^{2}}\left(1-\frac{\tan \left(\sqrt{\frac{p{l}^{2}}{16{EI}}}\right)}{\sqrt{\frac{p{l}^{2}}{16{EI}}}}+\frac{{\tan }^{2}\left(\sqrt{\frac{p{l}^{2}}{16{EI}}}\right)}{3}\right){F}^{2}-\frac{{p}_{1}}{{{y}_{{{m}}}\left(p-{p}_{1}\right)}^{2}}F+\frac{l\left(2p{p}_{1}^{2}-{p}^{2}{p}_{1}\right)}{16{EI}{\left(p-{p}_{1}\right)}^{2}}+\frac{pl}{12{G}^{2}{EI}}=0$$

### Fabrication and simple package

**Fig. 8:**
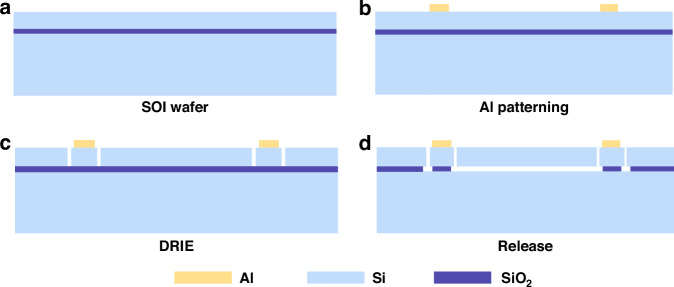
Fabrication process of the MEMS accelerometer. **a** SOI wafer with a low resistivity (0.01 Ω cm) and thick (25 μm) device layer. **b** Sputtering an Al film with a thickness of 800 nm and patterned by a mixture of H_3_PO_4_ (85%), H_3_NO_3_, CH₃COOH, and H_2_O at 45 °C for 2 min. **c** DRIE to pattern the accelerometer structure using the standard Bosch process for 7.2 min. **d** HF vapor release for at least 150 min to obtain the moveable structure of the accelerometer

The fabrication of the accelerometer die was conducted using an SOI wafer in the cleanroom at the Shanghai Institute of Microsystem and Information Technology (SIMIT). According to Eq. (12), the curved beam width and depth should be designed as small as possible to minimize bias force and displacement. Considering the resolution limits of the lithography system, the curved beam width was designed as 2 μm. Additionally, considering the stress limits of the curved beam and DRIE process stability, the depth (equivalent to device layer thickness) was selected to be 25 μm. A (100) silicon was selected due to its excellent mechanical strength, excellent surface flatness, and low defect density, which are critical for the fabrication of the proposed MEMS accelerometer. A SOI wafer with a highly doped device layer was selected due to its suitability for the fabrication of the MEMS capacitive accelerometer. Therefore, the accelerometer die was chosen to be fabricated on an SOI wafer with a low resistivity (0.01 $\Omega \cdot {\rm{cm}}$) and thick (25 ${\rm{\mu }}{\rm{m}}$) device layer, as shown in Fig. 8a. The fabrication steps involved photolithography, metal deposition, wet etching, DRIE, and HF vapor etching. The fabrication began with sputtering an Al film (800 nm thickness) on the device layer of the wafer, the Al film is then patterned by using a mixture of H_3_PO_4_ (85%), H_3_NO_3_, CH₃COOH, and H_2_O at 45 °C for 2 min, as shown in Fig. 8b. Next, the wafer was loaded into an SPTS DSi (SPTS Technologies Ltd.) to run a standard Bosch process for 7.2 min until the top layer of silicon was etched to the buried oxide layer, leaving behind the accelerometer structure including proof mass, comb fingers, curved beams, and release holes, as shown in Fig. 8c. Finally, the wafer was loaded into SPTS Monarch 3 (SPTS Technologies Ltd.) for at least 150 min to remove the buried oxide layer under the accelerometer structure, and the remaining buried oxide layer provides electrical isolation between electrodes, as shown in Fig. 8d.

A laser ablation dicing technique is used to eliminate the complications of protecting the moveable structure, which is necessary for saw dicing. After laser dicing, the accelerometer die was attached to a carrier PCB by applying a low-stress die attach adhesive to the bottom and curing it. The pads on the die and PCB were wire bonded using gold wires to achieve the electrical connection.

### Interface circuit and experiments setup

The interface circuit includes a capacitance-to-voltage converter (CVC) and a voltage amplifier. The CVC consists of a single-ended charge sensing amplifier, a demodulator, a DC-block, and a low-pass filter (LPF). As shown in Fig. 6a, an AC signal is converted into two differential carrier signals with the same amplitudes but an opposite phase through a high-speed differential driver AD8131, namely *V*_c_+ and *V*_c_−. The single-ended charge sensing amplifier (CSA) includes a half-bridge consisting of the differential capacitors *C*_1_ and *C*_2_, a high-precision amplifier AD8610, an integrating capacitor *C*_f_ and a feedback resistor *R*_f_. When an acceleration input is applied to the accelerometer, ${{C}}_{1}={{C}}_{0}+\Delta {C}$ and ${{C}}_{2}={{C}}_{0}-\Delta {C}$. If the frequency of the carrier signals is sufficiently large (${\omega }{{R}}_{{\rm{f}}}{{C}}_{{\rm{f}}}\gg 1$), the output voltage of the single-ended charge sensing amplifier (*V*_out_) can be written as^51^

37 $${{V}}_{{\rm{out}}}=-\frac{2\Delta C}{{C}_{{\rm {f}}}}{V}_{{\rm {c}}}$$

The output voltage of CSA linearly varies with the acceleration input. The acceleration signal is modulated to the high-frequency domain, which can significantly suppress the low-frequency domain noises in the readout circuit. The modulated signal is demodulated using a high-precision synchronous demodulator AD630, and the acceleration signal is recovered to the low-frequency domain. The DC component and high-frequency noises of the demodulated signal are suppressed through a DC block and an LPF. Then, the signal is amplified using a voltage amplifier.

The interface circuit outputs a voltage proportional to the applied acceleration in the accelerometer sensing direction. The input amplitude and frequency of the applied acceleration are controlled by a commercial shaker (B&K 3629). The signal generator (SIGLENT SDG6052X-E) is used to provide an external carrier signal for the readout circuit, and the DC power sources (Agilent E3631A) are used to provide the bias voltage (*V*_b_) and power the amplifiers. The output signal of the accelerometer is transmitted real-time data to a PC through the LAN-XI data acquisition module (B&K 3629).
